# Track Structure Components: Characterizing Energy Deposited in Spherical Cells from Direct and Peripheral HZE Ion Hits

**DOI:** 10.3390/life11111112

**Published:** 2021-10-20

**Authors:** Ianik Plante, Floriane Poignant, Tony Slaba

**Affiliations:** 1 KBR, Houston, TX 77058, USA; 2 National Institute of Aerospace, Hampton, VA 23666, USA; floriane.a.poignant@nasa.gov; 3 NASA Langley Research Center, Hampton, VA 23681, USA; tony.c.slaba@nasa.gov

**Keywords:** radiation track structure, spherical targets, energy deposition, heavy ions, HZE, ionizing radiation

## Abstract

To understand the biological effects of radiation, it is important to determine how ionizing radiation deposits energy in micrometric targets. The energy deposited in a target located in an irradiated tissue is a function of several factors such as the radiation type and the irradiated volume size. We simulated the energy deposited by energetic ions in spherical targets of 1, 2, 4, and 8 µm radii encompassed in irradiated parallelepiped volumes of various sizes using the stochastic radiation track structure code Relativistic Ion Tracks (RITRACKS). Because cells are usually part of a tissue when they are irradiated, electrons originating from radiation tracks in neighboring volumes also contribute to energy deposition in the target. To account for this contribution, we used periodic boundary conditions in the simulations. We found that the single-ion spectra of energy deposition in targets comprises two components: the direct ion hits to the targets, which is identical in all irradiation conditions, and the contribution of hits from electrons from neighboring volumes, which depends on the irradiated volume. We also calculated an analytical expression of the indirect hit contributions using the local effect model, which showed results similar to those obtained with RITRACKS.

## 1. Introduction

Ionizing radiation, especially ions, is unique because it transfers energy on an atomic scale in a highly concentrated manner. The average energy absorbed per unit mass of irradiated medium is quantified using the absorbed (macroscopic) dose. At micrometric scales, the energy deposited by ionizing radiation is highly non-homogeneous due to the radiation track structure [1]. Thus, while absorbed dose is a global quantity in the determination of radiation effects, the patterns of energy deposition provide a deeper understanding and basis for estimating the responses to ionizing radiation.

Microdosimetry is the study and quantification of the spatial and temporal distribution of absorbed energy in irradiated matter volumes of dimensions in the micrometer range, such as cells or cellular organelles [2]. It focuses on the heterogeneous and stochastic nature of radiation and provides concepts and methods for measuring the associated physical quantities [3,4,5]. Although microdosimetry concepts can be used to interpret a variety of radiation effects such as single event upsets in integrated circuits, its principal applications have been intended for understanding basic mechanisms of biological response. Microdosimetry has been used to estimate quality factors for risk assessment in radiation protection [6,7] and to quantify relative biological effectiveness using the microdosimetric kinetic model for planning treatments using hadron radiation therapy [8,9].

Previously, calculations were made using the Monte Carlo track structure simulation code Relativistic Ion Tracks (RITRACKS) to obtain single-ion spectra for irradiation by ions to compare them to measurements made with tissue-equivalent proportional counters (TEPCs) [10], which are spherical chambers filled with a tissue-equivalent gas. These calculations were similar to those conducted in this study for spherical targets, but with a wall included. The simulation results were markedly different from the experimental results. However, the TEPCs that were considered for the simulations have a plastic wall of density of 1.127 g/cm^3^ and an internal gas at a density of 8 × 10^−5^ g/cm^3^. Therefore, the density of the wall is about 14,087 times greater than the density of the gas inside the target. At the tissue scale, because the simulated density is equal to water (1 g/cm^3^), the corresponding density of the wall would be 14,087 g/cm^3^. Simulating track structure in a medium of this density is problematic, and likely unrealistic. Indeed, the importance of wall effects in TEPCs has been known for decades; in general, the wall distorts the spectra of energy deposition and causes a certain shift of the spectra towards higher values [11]. Therefore, wall-less TEPCs were also built [12]. However, because of the material and density considerations, we did not simulate TEPCs for this work.

There are situations where neither the μm nor nm scale have been shown to play a predominant role in the contribution to the overall radiation damage [13]. In this work, we simulated energy deposition in wall-less spherical water targets whose radii (1 to 8 µm) are representative of cellular structures. These sizes are larger than dimensions of DNA (~2 nm), nucleosomes (~10 nm), and chromatin fiber (~25 nm) [14], but they compare to the size of human cell nuclei, which were used in several radiation models [15,16,17]. The targets were encompassed in volumes also filled with water and irradiated by ions of various types and energies, with linear energy transfer (LET) ranging from 0.22 to 149 keV/µm. These ions can generate secondary delta electrons that can have sufficient energies to travel beyond a few millimeters. Simulating such large volume with a stochastic track structure simulation code would be impractical. Therefore, we used periodic boundary conditions (PBCs) as a solution to account for energy deposition due to delta electrons generated by distant tracks. The method was validated by comparing our simulation results to theoretical and published results. We further calculated single-ion energy deposition spectra for different radiation types and target sizes.

## 2. Methods

### 2.1. Simulation of Volume Irradiation by Stochastic Radiation Tracks

Stochastic radiation tracks were simulated with the program RITRACKS, which was developed at NASA to simulate detailed stochastic radiation tracks. The basis of the code is described in [18] and in the references therein. Briefly, RITRACKS calculates the energy deposition events (ionizations and excitations of water molecules) induced by protons and high charge (Z) and energy (HZE) particles, as well as the energies, positions, and directions of the secondary electrons and their full transport in an irradiated volume. Additionally, RITRACKS can calculate energy deposition in voxels [19] as well as in spherical and cylindrical targets [20]. The deposited energy is calculated as follows for interaction events. For ionizations of external layers of the water molecule, the energy deposited corresponds to the potential energy of the orbital of the ionized electron. For the rare ionizations of the internal layer of the water molecule, a 500 eV Auger electron is generated, and the energy deposited locally is the potential energy (540 eV) minus the energy of the Auger electron (540 − 500 = 40 eV). For excitations, the energy deposited is determined by sampling of the differential cross section and corresponds to the energy loss by the incident particle. For low-energy electrons, which can end their trajectories by thermalization or dissociative attachment to a water molecule, the energy deposited is equal to the electron energy. Because the electrons are fully simulated until the end of their trajectories, energy conservation is verified, i.e., the sum of all energy deposited is equal to the initial electron energy.

**Figure 1:**
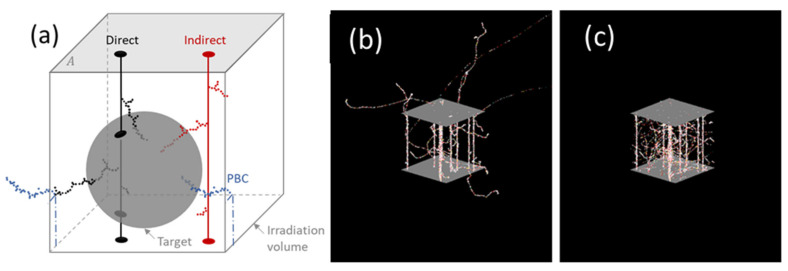
Scheme of RITRACKS calculations and the definition of direct/indirect contributions (**a**) and RITRACKS results for an irradiation of a volume by 25 MeV/n carbon ions, without (**b**) and with (**c**) PBCs. Using PBCs, it is possible for a delta electron to leave the volume many times; if it is the case, it will reenter as many times as necessary for all its energy to be spent deposited in the simulated volume.

As shown in Figure 1, one target was placed at the center of an irradiated volume (boxes) of equal size or larger. For this work, we used wall-less spherical targets of 1, 2, 4, and 8 µm radii. The volume was irradiated uniformly by a given ion type and energy along one direction, as in [21]. The number of tracks *n* impinging the volume in each simulation history was obtained by sampling from the Poisson distribution,

(1) $$p\left(n\right)=\frac{\lambda^{n}e^{-\lambda }}{n!},$$

where λ=ϕA is the average number of tracks, *A* is the area of the irradiated volume surface (the grey surface in Figure 1), and ϕ is obtained from a given dose D using the well-known equation,

(2) $$D\left(\mathrm{Gy}\right)=1.6\times 10^{9}\phi \left({\mathrm{cm}}^{-2}\right)\times \mathrm{LET}\left(\mathrm{keV}/\mu m\right).$$

The LET of a particle is calculated using Bethe’s equation, with corrections [22]. For sampling the Poisson distribution, an algorithm from the Numerical Recipes book [23] with appropriate modifications to use in the code was used.

The ion tracks are simulated within the irradiated volume, starting at the bottom surface, following a trajectory perpendicular relative to that surface, and followed until they leave the volume. The secondary electrons are fully simulated, even if they leave the irradiation volume or do not contribute to the energy deposition in the target. The ions considered for this work have relatively high energy (≥250 MeV/n). Therefore, the LET of the ions simulated does not vary much over a few microns, and the ions never stop in the irradiation volume.

### 2.2. Effects of the Irradiated Volume Size on Dose Calculations

Initial calculations using the simulation setup described in Figure 1 yielded target doses that were significantly and consistently lower than expected (as much as 23% lower). This is because many of the secondary electrons, or delta rays, from an ion track have sufficient energy to leave the irradiated volume, but compensating energy depositions from delta rays that originated in neighboring volume were not taken into account in the microdosimetric calculation. Indeed, Equation (2) applies when the irradiated volume is in electronic equilibrium, i.e., the flux of electrons leaving the volume is compensated by a flux of electrons generated in surrounding volumes and entering into it.

To reconcile the calculated dose with the expected dose, we initially attempted to increase the size of the irradiated area, assuming that most delta-ray contributions would be from tracks in the vicinity of the target. This approach was clearly insufficient because delta rays in the MeV range can travel up to 100 mm [24]. While the probability for a distant track to produce a delta ray that reach the target decreases with increasing distance, the differential irradiation area (dA=2πrdr) at a distance r from the target increases with r. As the number of tracks in the differential irradiation area is ϕdA, the possible contributions for more tracks must be considered, and the overall contributions of distant tracks are not negligible. Because simulating a volume of 100 mm is impractical, we used two approaches: the restricted LET for the calculation of the expected dose and periodic boundary conditions (PBCs) on the irradiated volume.

#### 2.2.1. Restricted LET

The restricted LET considers the extent of the penumbra of radiation tracks and the size of the region of interest, which is the target. In the Chatterjee–Schaefer approximation, the radially restricted LET (LET_Δ_) can be written as [25]:

(3) $${\mathrm{LET}}_{\Delta }=\frac{{\mathrm{LET}}_{\infty }}{2}\left[1+\frac{1+2\ln\left(r/r_{c}\right)}{1+2\ln\left(r_{p}/r_{c}\right)}\right],$$

where r is the radial distance (taken here as the radius of the sphere),

(4a) $$r_{c}=0.0116\beta ,$$

(4b) $$r_{p}=0.768E-1.925\sqrt{E}+1.257,$$

E is the energy per nucleon, β=v/c is the ratio between the velocity of the particle (v) and the speed of light (c), and ${\mathrm{LET}}_{\infty }$ is the LET value predicted by Bethe’s equation. By replacing LET by LET_Δ_ in Equation (2), the expected dose for a given fluence is lower.

The radially restricted LET implies a cylindrical symmetry along the axis of the track; therefore, the approximation that the tracks are centered with a radial extent of 1 µm is made when the dose is calculated inside a parallelepiped. However, because the energy deposition is the highest in the core region, the geometry effect is not too important.

#### 2.2.2. PBCs

PBCs are often used in computer simulations and mathematical models to approximate large (or infinite) systems using a small representative volume of space called the unit cell. The entire large space, which may or may not be periodic, is tiled with unit cells to approximate the real physical space. Due to the cylindrical symmetry of the radiation tracks interacting with a spherical target, we used a cylindrical irradiation volume with a disk as the irradiated surface in previous work. However, PBCs are not appropriate for use in this case because cylinders (as irradiation volumes) cannot tile the space entirely. Indeed, the calculated dose was incorrect in these situations [10].

When an electron passes through one side of the unit cell, it appears on the opposite side with the same velocity vector, as illustrated in Figure 1. Furthermore, every time an electron crosses a volume boundary, it is assumed to be originating from a different track, effectively mimicking the contribution of delta electrons originating from tracks in distant volumes. An example of a simulated irradiation of a volume by ^12^C^6+^ ion tracks obtained by RITRACKS with and without PBCs is shown in Figure 1. When PBCs are not applied (panel b), several electron tracks deposit a fraction of their energy outside the irradiation volume. When PBCs are applied (panel c), the energy of all electrons is fully deposited in the irradiated volume.

In principle it is possible that electrons from the neighboring volumes can carry more energy into the irradiated volume than the energy of the electrons that leaves the volume. In a simulation dose using PBCs, this does not happen as the energy of the electrons that is deposited inside comes from a given track and is conserved. There are several instances where electrons can reenter the irradiated volume many times. This mimics the delta-ray contributions from more distant neighboring volumes (i.e., from non-adjacent volumes).

### 2.3. Single-Ion Energy Deposition Spectra

We calculated the spectra of energy deposition for individual tracks, referred to herein as single-ion energy deposition spectra. For each simulation, n tracks are generated as explained in Section 2.1. The code RITRACKS was modified to assign an identification number to each track. As illustrated in Figure 1, the single-ion energy deposition spectra are composed of direct and indirect components. Therefore, three single-ion deposition spectra are obtained for each simulation: direct, indirect, and total. When the energy deposited from electrons originates from tracks whose axis crosses the target, it is considered a contribution to the direct component; otherwise, it is considered a contribution to the indirect component. All energy deposited in the target contributes to the total single-ion energy deposition spectra. All electron tracks that cross the edge of the volume and are re-inserted (PBCs) are considered contributions to the indirect component, as they mimic delta electrons generated by ion tracks from neighboring volumes. In addition, each time an electron crosses a boundary, a different track identification number is assigned to it because it is considered to be originating from a different track. It should be noted that the terminology used in this paper does not correspond to direct and indirect effects, which are used in radiation biology to describe the direct ionization of DNA (direct effect) and the reaction between a radiolytic species and DNA (indirect effect).

To obtain the single-ion energy deposition spectra for an irradiation dose D, we considered each individual track and scored its associated energy deposition by summing all the energy deposition events (ionizations, excitations, etc.) that occurred within the target. The same process was repeated for all tracks to obtain a distribution. As the average number of tracks is directly proportional to the dose, we normalized the single-ion energy deposition spectra so that its integral, when converted from energy to dose, corresponds to 1 Gy. The single-ion energy deposition spectra results were analyzed with adaptive width kernel density estimation (awKDE) [26]. The number of simulation histories was chosen to ensure convergence of the results. For an irradiation dose of 1 Gy, convergence of the results was achieved with a number of histories varying from 10^3^ (low LET, large targets) to 10^6^ (high-LET, small targets).

### 2.4. Calculation of the Indirect Dose Contribution Using the Local Effect Model

Many amorphous track models such as those from Kiefer and Straaten [27], Kobetich and Katz [28], Wang and Vassiliev [29], and the local effect model (LEM) assume that the radial dose is well approximated by a 1/*r*^2^ dependency in the penumbra region or in large parts of it [30]. For example, the radial dose used in the local effect model is,

(5) $$D_{p}\left(r\right)\;=\;\left\{\begin{array}{cc} \frac{\lambda \mathrm{LET}}{r_{min}^{2}} & r\leq r_{min}\\ \frac{\lambda \mathrm{LET}}{r^{2}} & r_{min}<r\leq r_{max}\\ 0 & r>r_{max} \end{array}\right.,$$

where *λ* is a normalization constant, equal to,

(6) $$\lambda =\frac{1}{\pi \rho \left[1+2\log\left(\frac{r_{max}}{r_{min}}\right)\right]}.$$

The core radius, $r_{min}$ = 0.0003 µm. The maximum radius, $r_{max}$, is determined by the electrons with the highest energy. It is given as $r_{max}=0.062\times E^{1.7}$, where $r_{max}$ is in µm, and E is the energy in MeV/n. The density ρ = 1 g/cm^3^ = 10^−15^ kg/µm^3^. Because the distances are given in µm, and the LET is usually given in units of keV/µm, the energy must be converted to J to obtain the dose in Gy. Therefore, the calculated result was multiplied by $f_{\;keV\;\to \;J}=1000\times 1.6\times 10^{-19}=1.6\times 10^{-16}\;\left(J/\mathrm{keV}\right)$.

For one track located outside a target of radius R, at distance $r_{0}>R$ from the center (impact parameter), the dose contribution to the target from this track can be calculated analytically (Supplementary Materials). The result is

(7) $$D_{sphere}=\frac{4\pi \lambda \mathrm{LET}}{V_{sphere}}\left\{R-\sqrt{r_{0}^{2}-R^{2}}\sin^{-1}\left(\frac{R}{r_{0}}\right)\right\}.$$

The dose contributions from tracks outside the target (from R to $r_{m}$) can be calculated by integrating the contributions of tracks located between $r_{0}$ and $r_{0}+dr_{0}$ outside the target. This calculation yields:

(8) $$D_{\mathrm{ind}}=\frac{2D}{R^{3}\left[1+2\log\left(\frac{r_{max}}{r_{min}}\right)\right]}\left\{R\left(r_{m}^{2}-R^{2}\right)\;+\;R^{3}\log\left(\frac{r_{m}}{R}\right)\;-\;{\left(r_{m}^{2}-R^{2}\right)}^{\frac{3}{2}}sin^{-1}\left(\frac{R}{r_{m}}\right)\right\}\;,$$

where D is the dose to the irradiated volume, and $r_{m}$ is a distance between the target radius R and the maximum electron range.

## 3. Results and Discussion

### 3.1. RITRACKS Microdosimetry Benchmark

We validated the RITRACKS results and the use of PBC by comparing RITRACKS predictions with theoretical calculation and published results.

#### 3.1.1. Comparison of Average Dose in Targets with Theoretical Predictions

**Figure 2:**
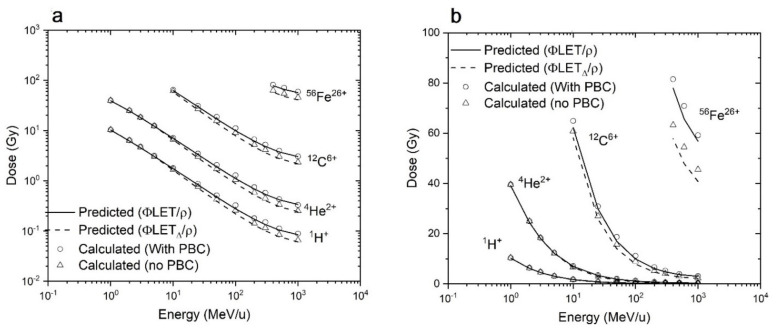
Predicted and calculated dose to a spherical target encompassed in the center of a cubical volume (2 × 2 × 2 µm) for one incident ion impinging the irradiated surface perpendicularly at a random location. Figures (**a**) and (**b**) are identical, but on different scales.

We simulated the irradiation of spherical targets of 1 µm radius and a box volume of 2 × 2 × 2 µm by one randomly incident single ion (i.e., fluence of 1 µm^−^^2^) and calculated the average dose imparted to the target. This box size is the smallest to encompass the target. Four types of ions were used: ^1^H^+^, ^4^He^2+^, ^12^C^6+^, and ^56^Fe^26+^. Energies varied from 1 to 1000 MeV/n. Calculations were performed both with and without PBCs. The results are shown in Figure 2.

Figure 2 shows that the calculated dose (without PBCs) is systematically lower than the predicted dose, $D\left(Gy\right)=1.6\times 10^{9}\phi \left({\mathrm{cm}}^{-2}\right)\times \mathrm{LET}\left(\mathrm{keV}/\mu m\right)$, and worsens at high energy per nucleon for all ions. This was observed previously in calculations of energy deposition in targets [31], and in other studies such as the simulation of chromosome aberrations [21]. As mentioned earlier, this happens because a significant portion of the energy is deposited outside of the irradiated volume. Indeed, in the relativistic collision theory, the maximum energy transferred from an ion of mass *M* and energy *E* to an electron of mass *m* is given by [32]:

(9) $$E_{Max}=\frac{2mc^{2}\left(\gamma^{2}-1\right)}{1+2\gamma \left(m/M\right)\;+\;{\left(m/M\right)}^{2}}\approx 4\frac{m}{M}E,$$

where γ is the relativistic quantity for the ion. For a 1000 MeV/n ion, $E_{Max}$ is about 3.33 MeV. An electron of this energy can travel several millimeters within tissues [24]. In Equation (3), the ratio of ${\mathrm{LET}}_{\Delta }/{\mathrm{LET}}_{\infty }$ is essentially a function of the energy per nucleon, which explains that the difference between the calculations with and without PBCs increases similarly with the energy per nucleon.

To reconcile the theoretical predictions with the calculations without PBC, we used the restricted LET (Equation (3)). As shown in Figure 2, the calculations of the energy deposition in spheres without PBCs is in very good agreement with the dose value predicted using the restricted LET. We may also note that the radially restricted LET implies a cylindrical symmetry along the axis of the track; therefore, the approximation that the tracks are centered with a radial extent of 1 µm is made when the dose is calculated inside a parallelepiped. However, because the energy deposition is the highest in the core, the geometry effect is not too important.

The calculations were repeated using PBCs on the irradiated volume, and the calculations are in much better agreement with the predicted dose calculated with ${\mathrm{LET}}_{\infty }$. While the calculations of predicted doses in targets using the two approaches (restricted LET or PBCs) are both similar to their simulation equivalent, the PBCs approach is preferred. This is because PBCs simulate the irradiation of a target in a tissue more realistically by considering electrons from neighboring volumes, whereas the radially restricted LET implies the intersection between a cylindrical track and a target.

#### 3.1.2. Comparison with Published Results

**Figure 3:**
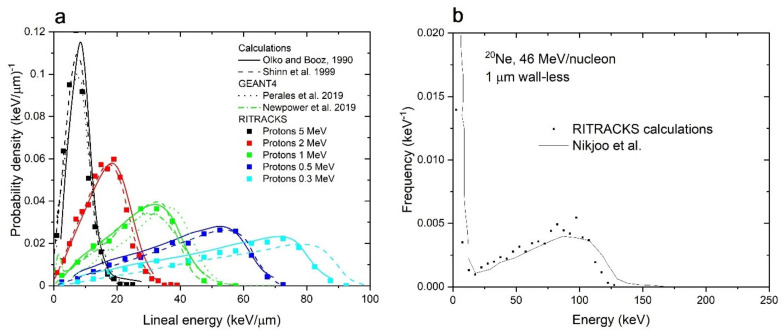
Comparison of simulation results from RITRACKS calculations and other codes. (**a**): probability distributions of lineal energy for a 1 µm diameter water sphere irradiated by a single randomly incident proton of 0.3, 0.5, 1.0, 2.0, and 5.0 MeV. The calculations made with RITRACKS (dots) are compared to those published in References [34,35,36,37] (lines). (**b**): Probability distribution of energy deposited in a 1 μm diameter water sphere irradiated by a single randomly incident Ne ion, 46 MeV/n (LET ~133 keV/μm).

The lineal energy, y, is defined as the quotient of the energy imparted, *ε*, to the matter in a volume from a single energy deposition event by a mean chord length, $\bar{l}$, in that volume. For a sphere of radius *R*, $\bar{l}=4R/3$, so that $y=\varepsilon /\bar{l}=3\varepsilon /4R$ [33]. Probability density distributions of lineal energy in a 1 μm diameter water sphere irradiated by a single randomly incident proton of 0.3, 0.5, 1, 2, and 5 MeV were calculated, and results are shown in Figure 3a. The energies were chosen to compare with results from older calculations [34,35], which are also shown in this figure. Contrary to other calculations performed in this work, the irradiated surface for the simulations shown in Figure 3a is a disk with radius equal to the target radius, which means that no tracks are generated outside the spherical volume. In general, the simulation results by RITRACKS are in good agreement with previous calculations made with an analytical model of Shinn et al. [34], Monte Carlo simulations from Olko and Booz [35], and recent GEANT4 simulations [36,37].

A similar calculation was performed for a 1 μm diameter water sphere irradiated by a single randomly incident Ne ion of 46 MeV/n. This is identical to the simulation conditions used by Nikjoo et al. [31]. For this simulation, the irradiated surface is a square (i.e., the spherical target is embedded in a cubical volume). The energy deposition spectrum in Figure 3b is similar to that in Figure 3a, except there is the peak at low energy for the Ne ion exposure. This peak is due to the energy deposited from tracks outside the volume. This peak is present in both RITRACKS and Nikjoo’s calculations. In general, Figure 3 shows that RITRACKS calculations are in excellent agreement with previous similar calculations for wall-less spheres.

### 3.2. Single-Ion Energy Distribution Spectra

#### 3.2.1. Direct and Indirect Contributions

**Figure 4:**
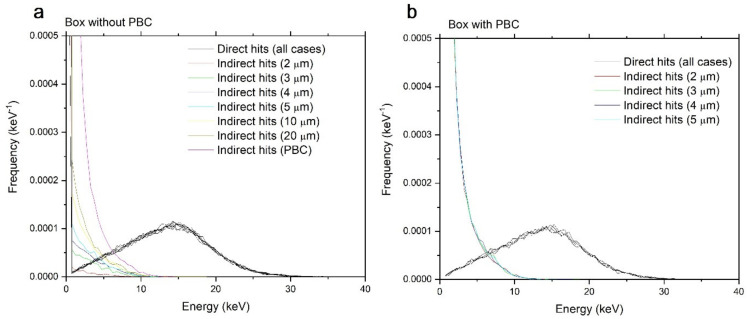
Calculation of energy deposition per track in spherical targets of radius of 1 µm by 1 Gy of 290 MeV/n carbon ions. The irradiation volumes were cubes of different sizes (2, 3, 4, 5, 10, and 20 µm), without PBCs (**a**) and with PBCs (**b**).

We simulated irradiation of a spherical target of 1-micron radius by 1 Gy of 290 MeV/n carbon ions. This ion and energy were chosen because of their relevance in space radiation and hadron therapy. The irradiation volumes were cubes whose lengths varied from 2, 3, 4, 5, 10, and 20 µm, with and without PBCs. The results are shown in Figure 4.

The most striking result in Figure 4 is that the direct hits component is identical regardless of the size of the irradiated volume or the presence or absence of PBCs. When PBCs are used, the indirect component is also identical for all sizes of irradiated volumes. Without PBCs, however, the indirect component depends on the size of the irradiated volume and converges asymptotically with increasing irradiated volume size towards the results obtained with PBCs. The direct and indirect contributions are given in Table 1 and Table 2. As the calculated result varies for each simulation history, the numbers shown here are averages over 10,000 simulations. The numbers have been rounded to 1 eV.

The results in Table 1 and Table 2 mostly confirm quantitatively that energy deposition in targets is the sum of the contributions from direct and indirect hits. The contribution of direct hits is mostly identical, regardless of the size of the box or the use of PBCs. The contribution of indirect hits varies with the size of the box when PBCs are not used.

For 290 MeV/n carbon ions, the maximum energy transfer to an electron (Equation (7)) is about 728 keV. The range of an electron of this energy is about 3.6 mm [24]. From geometric considerations, calculation time as well as memory requirements scale roughly with the box dimension cubed. Increasing the irradiated volume by a factor of 10 increases the calculation time by a factor of at least 1000. Therefore, simulating a volume in the range of a millimeter is practically impossible. However, this work shows that simulating such a large volume is not necessary because convergence is already reached for the smallest irradiation box when PBCs are used.

#### 3.2.2. Variation with Dose, Radius, and LET

The initial calculations were carried out as a function of dose. For a simulation with a given ion type, energy, target radius, irradiated volume, and boundary conditions (PBCs on/off), as the number of tracks is directly proportional to the dose (Equation (2)), increasing the dose is equivalent to increasing the number of tracks. Because the calculated quantity is the single-ion spectra, all tracks are independent; therefore, increasing the dose does not change the shape of the spectra except reducing statistical fluctuations.

**Figure 5:**
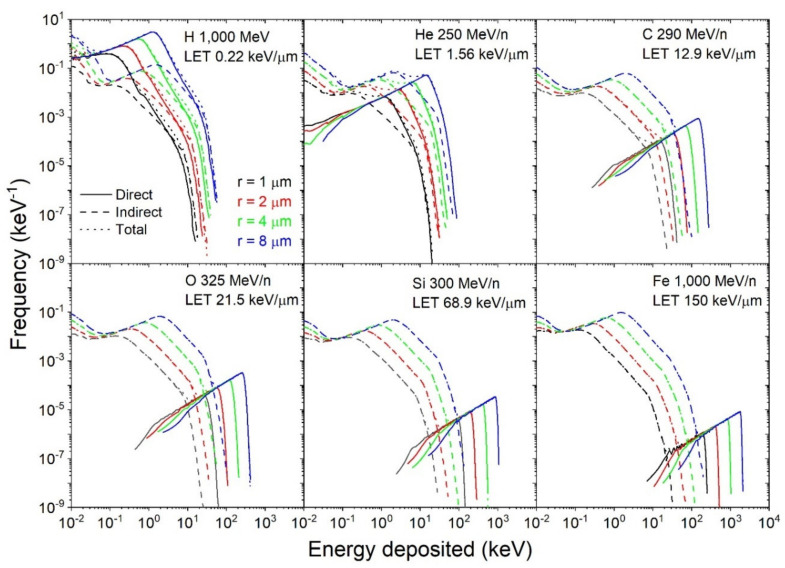
Single-ion energy deposition spectra for different ions (1000 MeV protons, 250 MeV/n helium, 290 MeV/n carbon, 325 MeV/n oxygen, 300 MeV/n silicon, and 1000 MeV/n iron ions) and target radii (1, 2, 4, and 8 µm). Contributions: direct (– –), dashed (- - -), total (⋅ ⋅ ⋅). Due to the large differences between the direct and indirect contributions at almost all energies, the total contributions overlap with the direct or indirect contribution, whichever is largest.

Additional calculations were performed as a function of the target radius (1, 2, 4, and 8 µm) for 1000 MeV protons, 250 MeV/n helium, 290 MeV/n carbon, 325 MeV/n oxygen, 300 MeV/n silicon, and 1000 MeV/n iron ions. The results are shown in Figure 5, with each panel showing the different contributions for a fixed beam and various target radii. The normalization is as follows:

(10) $$k\int_{0}^{\infty }\varepsilon f\left(\varepsilon \right)d\varepsilon =D,$$

where $f\left(\varepsilon \right)$ represents the probability density of energy deposition per single-ion track, normalized to the irradiation dose D (i.e., divided by the dose *D*) and *k* = $1.6\times 10^{-19}\;\left(J\cdot {\mathrm{eV}}^{-1}\right)\times m_{T}\;\left(\mathrm{kg}\right)$ is a unit conversion factor, with $m_{T}$ the mass of the target.

#### 3.2.3. Scaling of the Single-Ion Spectra

**Figure 6:**
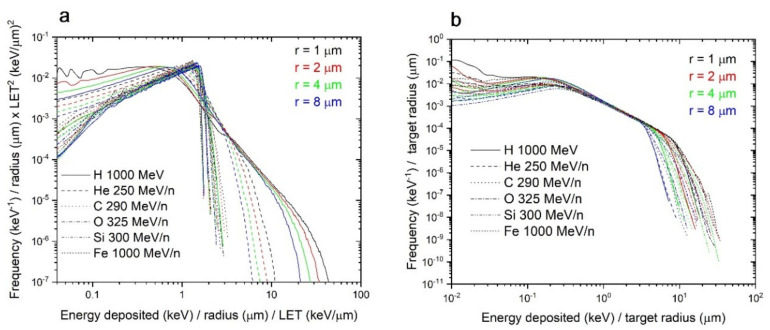
Scaling of the energy distribution spectra. (**a**) direct energy distribution spectra of all ions (1000 MeV protons, 250 MeV/n helium, 290 MeV/n carbon, 325 MeV/n oxygen, 300 MeV/n silicon, and 1000 MeV/n iron ions) and target sizes (1, 2, 4, and 8 µm), normalized to the LET and radius in the energy axis, and to LET^2^ and radius in the frequency axis. (**b**) indirect energy distribution spectra, normalized to the target radius.

The direct contribution spectra have similar shapes that scale with the radius, and with the LET. To put this in evidence, the direct contribution was replotted by dividing the energy and frequency axis by the target radius and multiplying by LET^2^. Indeed, the results look very similar (except for H and He) as shown in Figure 6a.

The direct contribution has a shape vaguely similar to a triangular distribution. This can be explained by considering the chord length *x* associated with a given value of r, in a sphere of radius R:

(11) $$x=2\sqrt{R^{2}-r^{2}}.$$

It can be shown that the chord length density distribution $p\left(x\right)$ in a sphere of radius R is

(12) $$p\left(x\right)\;=\;\left\{\begin{array}{c} \begin{array}{cc} \frac{x}{2R^{2}} & x\leq 2R \end{array}\\ \begin{array}{cc} 0 & x>2R \end{array} \end{array}\right..$$

The distribution $p\left(x\right)$ is normalized to 1, as expected:

(13) $$\int_{0}^{2R}p\left(x\right)dx=1.$$

The average chord length value $\bar{l}$ is given by

(14) $$\bar{l}=\int_{0}^{2R}xp\left(x\right)dx=\;\frac{4}{3}R.$$

As the energy deposited for perfectly linear tracks (i.e., tracks where energy is deposited uniformly along the track axis) would be equal to E=LET·x, the energy distribution in a sphere is

(15) $$p_{sphere}\left(E\right)\;=\;\left\{\begin{array}{cc} \frac{E}{2R^{2}{\mathrm{LET}}^{2}} & E\leq 2R\mathrm{LET}\\ 0 & E>2R\mathrm{LET} \end{array}\right..$$

This is a right-angle triangular distribution. However, since the track structures are not perfectly linear, the shape of the direct contribution deviates from this model. This appears to be particularly true for low-LET tracks. This is likely due to the proportion of the energy deposition by secondary electrons rather than the primary ion at low LET.

**Figure 7:**
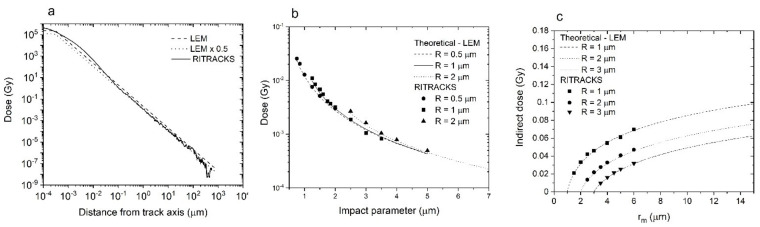
Comparison of analytical calculations using the LEM and results from RITRACKS simulations. (**a**) radial dose for a carbon ion, 290 MeV/n, calculated by RITRACKS, the LEM (Equation (3)) and the LEM with a factor 0.5 applied. (**b**) dose to spherical targets as a function of the impact parameter for one single 290 MeV/n carbon track (dots), and LEM prediction (lines), for targets of radius 0.5, 1, and 2 μm. (**c**) calculations of indirect dose in targets of radius 1, 2, and 3 μm, calculated by RITRACKS (dots) and compared with analytical LEM calculations (lines). For this simulation, a disk surface of radius rm was irradiated uniformly by 290 MeV/n carbon tracks. For the LEM results, a radial dose multiplicative factor of 0.5 was applied. The calculations were conducted without PBC.

The radial dose profile trends given by RITRACKS simulations and by the LEM are similar, especially for distances to the track axis greater than 0.1 μm (Figure 7a). However, the radial dose calculated by RITRACKS is consistently smaller than the LEM at most distances greater than 0.1 μm. Therefore, we multiplied the LEM values by 0.5 in the calculations shown below. It is possible to do so because difference in the radial dose profiles between RITRACKS and the LEM is limited to distances greater than ~0.1 µm, so that the influence on the target dose calculations (Equations (7) and (8)) is negligible.

We calculated the dose to the target using RITRACKS from tracks at different impact parameters and compared the results with the LEM predictions (Equation (7)). PBCs were not used in the simulations because of the geometry used to obtain Equation (7). The results are shown in Figure 7b. Using Equation (8), we also calculated the dose to a target from tracks at distances $R<r_{m}<r_{max}$ to simulate the indirect contributions. The results are compared to the stochastic track simulations (Figure 7c). Again, the calculations of the LEM-based analytical model and RITRACKS are mostly identical.

**Figure 8:**
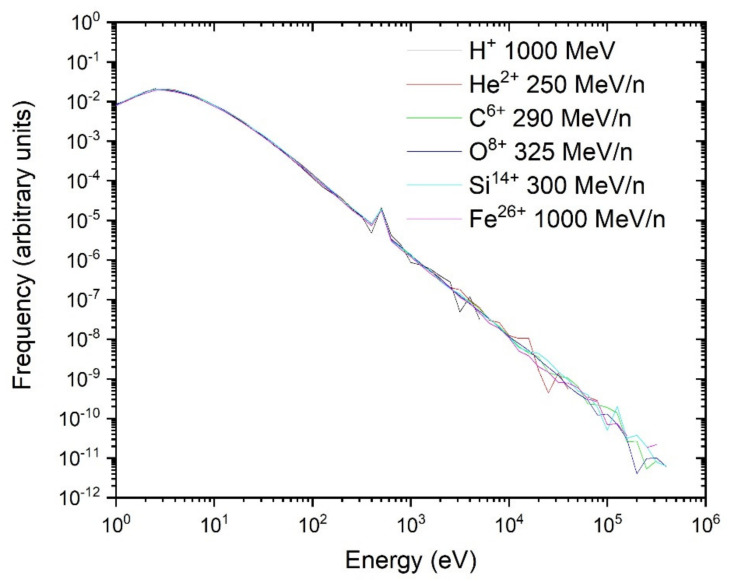
Ejected electron energy spectra for all ions, normalized to the LET. The peak at 500 eV corresponds to Auger electrons that are ejected when an internal orbital of the water molecule is ionized.

Contrary to direct contributions, for a given target size, the indirect contributions are mostly identical regardless of the LET. The number of electrons making indirect contributions are determined by the dose and LET of the initial tracks, but their energies are mostly dependent on the energy per nucleon. All ions used for the single-ion energy deposition spectra are relatively high energy (≥250 MeV/n); therefore, many electrons also have high energy. Indeed, the distribution of energy from ejected electrons is similar for all ions (Figure 8), although the number of ejected electrons is proportional to the LET of particles. Therefore, on an individual track basis, these electrons have mostly identical contribution to the target dose. Indeed, the indirect contributions of all ions are similar for each ion, when normalized for the target size (Figure 6b). The results shown in Figure 6b can be explained by considering that indirect hits come from electrons.

## 4. Conclusions and Perspectives

To understand the biological effects of ionizing radiation, it is important to consider the energy deposition in target volumes representative of cellular structure. In general, the results of our simulations agree with published results of simulations made using different codes. Our calculations show that the dose spectrum from a single ion is given by the sum of two components—direct and indirect contributions. Furthermore, our stochastic simulation results compare to analytical predictions of the LEM.

The simulations performed in initial work raised two concerns. The first concern is that the dose recorded in the target is lower than expected when PBCs were not used. This is explained by electrons escaping the irradiated volume and the lack of compensation from electrons leaking into the target from neighboring volumes. Because the dimension of the irradiated area is usually well beyond a cm, cells irradiated in tissues are usually at charge equilibrium, which means that energy loss due to delta electrons leaving cells is compensated by energy gain from electrons entering cells. Track structure calculations cannot go beyond dimensions of tens of µm to keep computing time reasonable. To address this, we used two approaches: the restricted LET to calculate the expected dose and PBCs. The latter is more realistic to model charge equilibrium. The second concern is thus whether the use of PBCs is an adequate approach or not. Our work has shown that the indirect contributions increase with increasing volume size, and that they eventually converge to values obtained by simulations using PBCs, which means that use of PBCs is an adequate approach to simulate the contributions of electrons from tracks in neighboring volumes.

This work validates the PBC approach for simulating energy deposition in targets. The PBC approach also provides a relatively easy way to significantly reduce the calculation time, and to perform simulations that would be difficult or impossible to do otherwise. Not only are large irradiation volumes computationally difficult and impractical to simulate, increasing the irradiation volume does not provide much new information. Indeed, in the calculation shown in Figure 4, increasing the size of the irradiation volume from 2 to 20 μm is insufficient to reach convergence to the PBCs value for indirect hits despite an increase in the calculation time by a factor of 1000. Ultimately, the indirect dose contribution will converge towards values calculated using PBCs, which can be performed considerably faster. This work shows that if PBCs are used, the code must account for the delta rays that cross boundaries belonging to a different track.

The understanding of how single-ion spectra are determined by the direct and indirect contributions, as well as the scaling by the target radius and LET, could provide some important insight into radiation-induced DNA damage, as the DNA is considered to be a target for radiation. This model could eventually be applied to understand the biological effects of ionizing radiation, notably in models involving DNA repair, such as those in [38,39,40,41]. With regard to DNA damage, it would be interesting to see whether the scaling of the single-ion spectra also applies to targets of nanometric sizes. In future work, we intend to use this approach and investigate the effect of radiation transport in digital mice to better represent radiation environment in vivo cells experience in ground-based experiments [42], and for microdosimetry calculation for realistic Galactic Cosmic Ray beams with or without shielding. This will allow us to study the impact of secondary particles produced in tissues or shielding and thus change of radiation quality on energy deposition in cells. We will use the Monte Carlo tool RITCARD [21] to calculate chromosome aberrations and correlate energy deposition in cell nuclei with measurable biological endpoints.

## Figures and Tables

**Table 1:** Calculations of the direct, indirect, and total energy deposition in spherical targets of radius 1 µm for 290 MeV/n carbon ion beam. The expected total dose is 1 Gy. Simulation without PBCs.

Box Size	Direct		Indirect		Sum	
µm	eV	Gy	eV	Gy	eV	Gy
2	20,175	0.7707	191	0.0073	20,366	0.7780
3	20,156	0.7700	691	0.0264	20,847	0.7963
4	20,448	0.7811	1001	0.0382	21,449	0.8194
5	20,393	0.7790	1159	0.0443	21,552	0.8233
10	20,566	0.7856	1612	0.0616	22,178	0.8472
20	20,794	0.7943	1872	0.0715	22,666	0.8658

**Table 2:** Calculation of the direct, indirect, and total energy deposition in spherical targets of radius 1 µm for 290 MeV/n carbon ion beam. The expected total dose is 1 Gy. Simulation with PBCs.

Box Size	Direct		Indirect		Sum	
µm	eV	Gy	eV	Gy	eV	Gy
2	19,603	0.7488	5885	0.2248	25,489	0.9737
3	20,146	0.7700	6071	0.2319	26,217	1.0015
4	20,258	0.7739	6192	0.2365	26,540	1.0138
5	20,328	0.7765	6140	0.2345	26,470	1.0111

## Data Availability

The simulation results can be obtained by request to the corresponding author. The software RITRACKS that has been used to perform these calculations is available at https://software.nasa.gov (accessed on 13 October 2021).

## Supplementary Materials

The following are available online at https://www.mdpi.com/article/10.3390/life11111112/s1, Figure S1: 3D view of an ion track and a spherical target. Figure S2: View of the plan XY of an ion track and a spherical target. Figure S3: Dose to target by multiple tracks.
