# Supplementary material for: Track Structure Components: Characterizing Energy Deposited in Spherical Cells from Direct and Peripheral HZE Ion Hits
Source: Life (Basel). 2021 Oct 20;11(11):1112. doi: 10.3390/life11111112 (PMC8619431; doi:10.3390/life11111112)
Supplement: Supplementary file 1 [file life-11-01112-s001.zip › life-1413506-supplementary.pdf]

life

MDPI

Supplementary materials

Calculation of the energy deposition by ion tracks outside the target

Ianik Plante, Floriane Poignant and Tony Slaba

1. The amorphous track model

Many amorphous track models assume that the radial dose has a $1/r^2$ dependency in the penumbra region (Elsässer et al. N. J. Phys. 10, 075005). For example, the dose used in the Local Effect Model (LEM) is

$$D_p(r) = \begin{cases} \frac{\lambda LET}{r_{min}^2} & r \le r_{min} \\ \frac{\lambda LET}{r^2} & r_{min} < r \le r_{max} \\ 0 & r > r_{max} \end{cases},$$

where λ is a normalization constant, equal to,

$$\lambda = \frac{1}{\pi\rho[1 + 2\log(r_{max}/r_{min})]}.$$

Furthermore, r_{min} = 0.0003 μm. The maximum radius r_{max} is determined by the electrons with the highest energy. It is given as $r_{max} = 0.062 \times E^{1.7}$, where r_{max} is in μm, and E is the energy in MeV/n. The density ρ = 1 g/cm^3 = 10^{-15} kg/μm^3. Since the distances are given in μm, and the LET is usually given in units of keV/μm, the energy should be converted to J to get the dose in Gy. Therefore, the calculated result should be multiplied by $f_{keV \to J} = 1{,}000 \times 1.6 \times 10^{-19} = 1.6 \times 10^{-16}\ (J/keV)$.

To ensure that the dose is properly calculated, the LEM track model was compared to the radial dose for a carbon ion, 290 MeV/n, calculated by RITRACKS. Results are shown in Figure 7a of the main article.

2. Calculation of the energy deposited in the sphere by one track

We would like to calculate the energy deposited in a spherical volume entirely located in the penumbra. This is illustrated in Figure Supp.1.

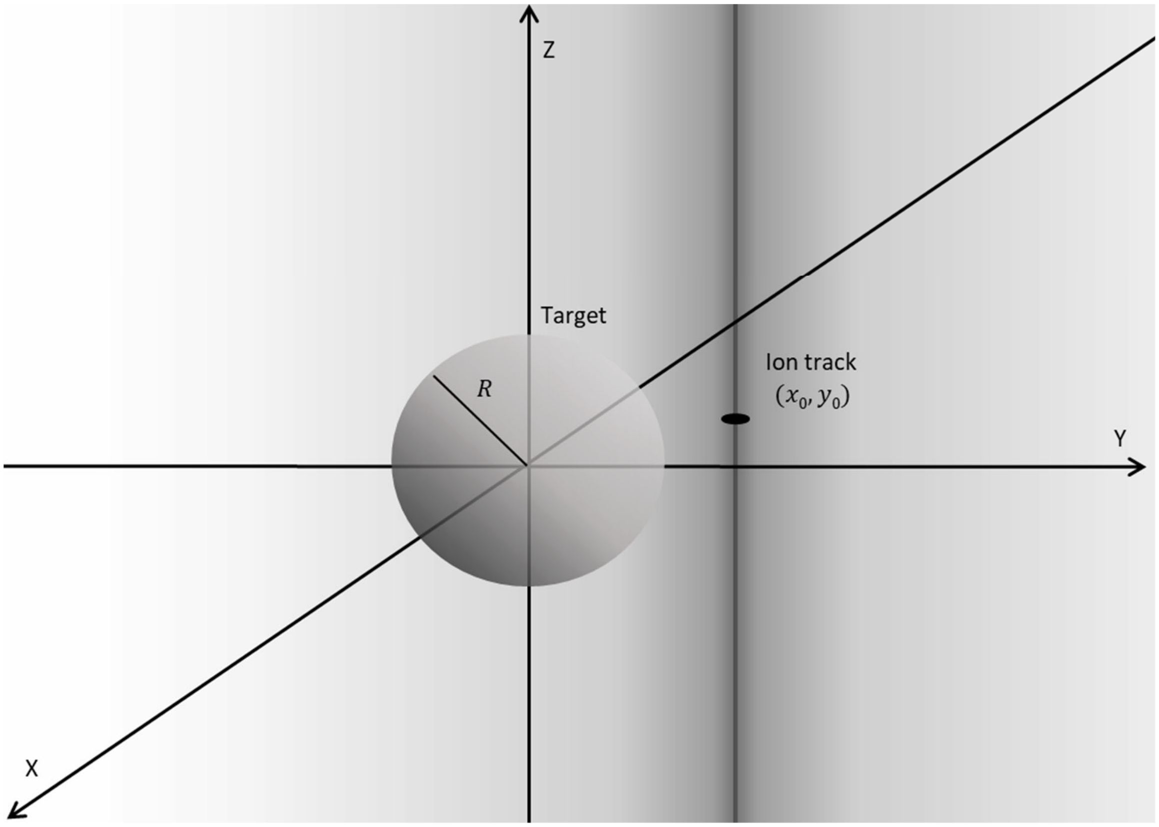

Figure S1. 3D view of an ion track and a spherical target.

The center of the track is located at (x_0, y_0), outside the sphere, and assumed to be parallel to the Z axis. R is the target radius. For any point (x, y, z) in the target, we have,

$$D_{sphere} = \frac{1}{V_{sphere}} \int_0^{2\pi} \int_0^R \int_{-\sqrt{R^2-r^2}}^{\sqrt{R^2-r^2}} f(x,y,z)\, r \, \mathrm{d}z\mathrm{d}r\mathrm{d}\theta \,,$$

where $f(x, y, z)$ is the dose at point (x, y, z). In the case of a radial dose and assuming the track is oriented along the Z axis, for any coordinate z we have,

$$f(x,y,z) = f(d) = \frac{D_0}{d^2} = \frac{D_0}{(x-x_0)^2 + (y-y_0)^2},$$

with $D_0 = \lambda LET$ in the LEM framework. As illustrated in Figure Supp.2, d is the distance between the track of coordinates (x_0, y_0) in the plan XY and the point (x, y). $R_p = \sqrt{R^2 - z^2}$ is the radius of the target in the given plane.

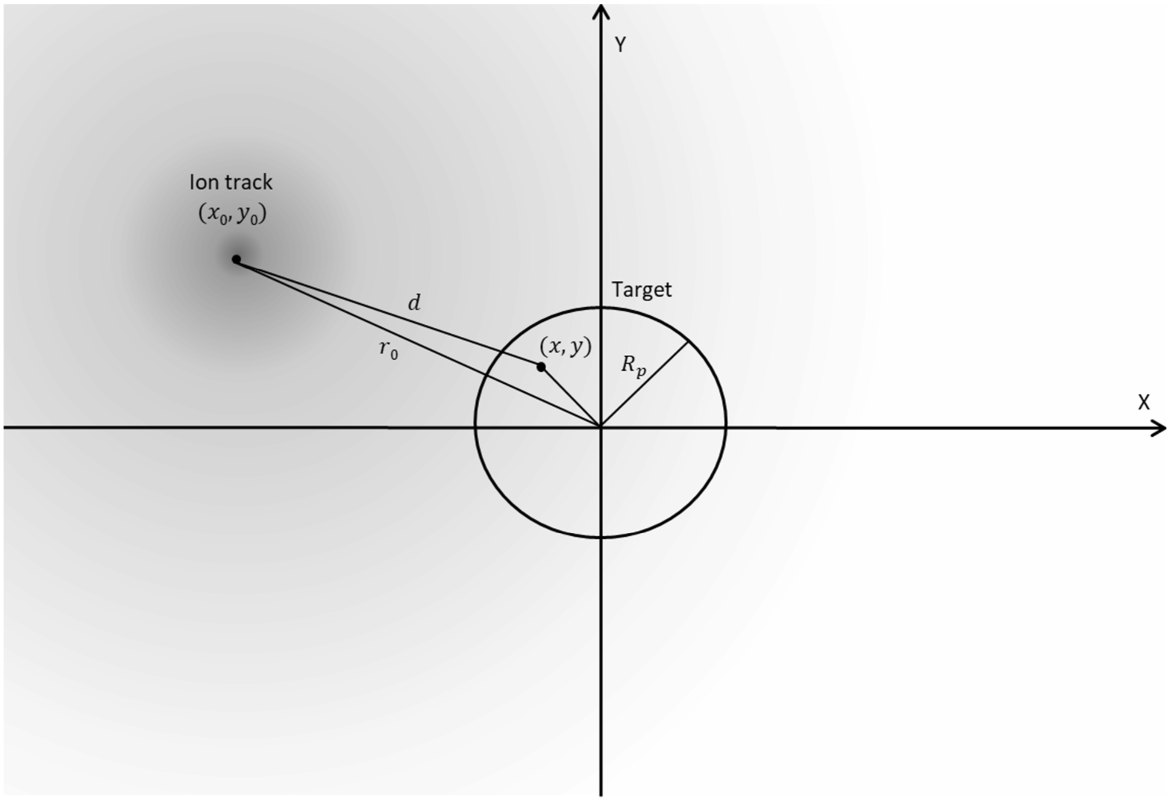

Figure S2. View of the plan XY of an ion track and a spherical target.

Using cylindrical coordinates $x = rcos\theta,\ y = rsin\theta,\ x_0 = r_0cos\theta_0,\ y_0 = r_0sin\theta_0$, we get

$$f(x, y, z) = \frac{D_0}{(rcos\theta - r_0cos\theta_0)^2 + (rsin\theta - r_0sin\theta_0)^2},$$

which simplifies to

$$f(x, y, z) = \frac{D_0}{r^2 + r_0^2 - 2rr_0\cos(\theta - \theta_0)}.$$

Since the problem is symmetric, we chose $\theta_0 = 0$ to further simplify. Hence, the integral to evaluate is

$$D_{sphere} = \frac{1}{V_{sphere}} \int_0^{2\pi} \int_0^R \int_{-\sqrt{R^2-r^2}}^{\sqrt{R^2-r^2}} \frac{D_0}{r^2 + r_0^2 - 2rr_0\cos\theta}\ r\ \mathrm{d}z\mathrm{d}r\mathrm{d}\theta\,.$$

Integrating over z, we find,

$$D_{sphere} = \frac{2D_0}{V_{sphere}} \int_0^{2\pi} \int_0^R \frac{r\sqrt{R^2 - r^2}}{r^2 + r_0^2 - 2rr_0\cos\theta}\ \mathrm{d}r\mathrm{d}\theta.$$

$$= \frac{2D_0}{V_{sphere}} \int_0^R r\sqrt{R^2 - r^2} \int_0^{2\pi} \frac{1}{r^2 + r_0^2 - 2rr_0\cos\theta}\ \mathrm{d}\theta\mathrm{d}r\,.$$

Using the Mathematica© software, we have:

$$\int_0^{2\pi} \frac{\mathrm{d}\theta}{r_0^2 + r^2 - 2rr_0cos\theta} = \frac{2\pi}{r_0^2 - r^2}.$$

Therefore,

$$D_{sphere} = \frac{4\pi D_0}{V_{sphere}} \int_0^R \frac{r\sqrt{R^2 - r^2}}{r_0^2 - r^2}\ \mathrm{d}r.$$

This can also be integrated analytically:

$$D_{sphere} = \frac{4\pi D_0}{V_{sphere}}\left\{R - \sqrt{r_0^2 - R^2}\sin^{-1}\left(\frac{R}{r_0}\right)\right\}.$$

Replacing $V_{sphere} = 4\pi R^3/3$ and $D_0 = \lambda LET$, we have,

$$D_{sphere}(Gy) = \frac{3 \times f_{keV\to J}\, \lambda LET}{R^3}\left\{R - \sqrt{r_0^2 - R^2}\sin^{-1}\left(\frac{R}{r_0}\right)\right\},$$

where r_0 and R are in µm, LET is in keV/µm, λ is in µm³/kg, and $f_{keV \to J} = 1.6 \times 10^{-16}\, J/keV$. Or equivalently

$$D_{sphere}(Gy) = \frac{3 \times f_{keV\to J}\, LET}{R^3\pi\rho[1 + 2\log\,(r_{max}/r_{min})]}\left\{R - \sqrt{r_0^2 - R^2}\sin^{-1}\left(\frac{R}{r_0}\right)\right\}.$$

Calculations of the dose to sphere as a function of the impact parameters comparing the results of stochastic track calculations by RITRACKS and the last equation are shown in Figure 7b of the main article.

3. Calculation of dose to the target from a uniform track field

Now that the contribution of one track has been calculated, we assume that the radiation tracks are uniformly distributed around the target.

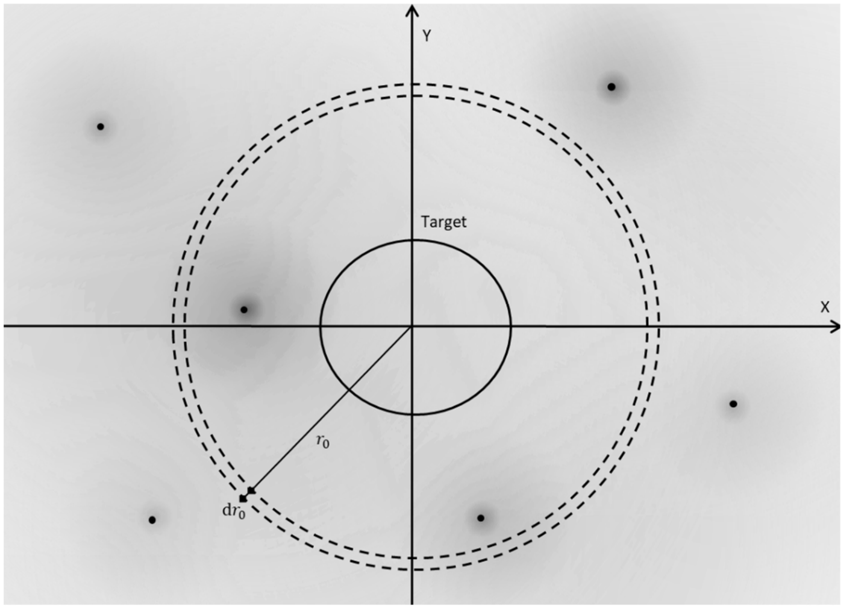

Figure S3. Dose to target by multiple tracks.

We now need to calculate the contribution of all tracks surrounding the target. Assuming a fluence of tracks ϕ, the number of tracks between r_0 and $r_0 + \mathrm{d}r_0$ (with $r_0 > R$) is (Figure Supp.3):

$$[\pi(r_0 + dr_0)^2 - \pi r_0^2]\phi = \phi 2\pi r_0 \mathrm{d}r_0.$$

The dose deposited by tracks between r_0 and $r_0 + \mathrm{d}r_0$ (in Gy) is the number of tracks multiplied by the dose per track, that is:

$$\phi 2\pi r_0 \mathrm{d}r_0 D_{sphere}(r_0) = \phi 2\pi r_0 \mathrm{d}r_0 \frac{4D_0\pi}{V_{sphere}}\left\{R - \sqrt{r_0^2 - R^2}sin^{-1}\left(\frac{R}{r_0}\right)\right\}.$$

To calculate the contribution from tracks up to r_m, where $R < r_m < r_{max}$, we integrate the latter:

$$D_{\text{ind}} = \int_R^{r_m} \phi 2\pi r_0 D_{sphere}(r_0)\mathrm{d}r_0 = \frac{\phi 8\pi^2 D_0}{V_{sphere}}\int_R^{r_m} r_0\left\{R - \sqrt{r_0^2 - R^2}sin^{-1}\left(\frac{R}{r_0}\right)\right\}\mathrm{d}r_0.$$

Using the Mathematica© software, we obtained an analytical result,

$$D_{\mathrm{ind}} = \frac{\phi 8\pi^2 D_0}{3V_{sphere}}\left\{R(r_m^2 - R^2) + R^3\log(r_m/R) - (r_m^2 - R^2)^{\frac{3}{2}} sin^{-1}\left(\frac{R}{r_m}\right)\right\}.$$

Let now consider the factor before the parenthesis. By the LEM,

$$D_0(Gy) = \lambda(\mu m^3/kg)\ LET\ (keV/\mu m)\ f_{keV\to J}\ (J/keV).$$

Besides, the dose $D(Gy)$ is related to the fluence and LET as follows:

$$D(Gy) = \frac{\phi(\mu m^{-2}) LET\ (keV/\mu m) f_{keV\to J}\ (J/keV)}{\rho(kg/\mu m^3)}.$$

So that $\phi D_0 = D(Gy)\ \lambda(\mu m^3/kg)\ \rho(kg/\mu m^3)$. Therefore,

$$\frac{8\ \pi^2\phi D_0}{3V_{sphere}} = \frac{8\pi^2\phi D_0}{3(4/3\pi R^3)} = \frac{2\pi\ \phi D_0}{R^3} = \frac{2\pi\ D\ \rho\lambda}{R^3}.$$

So we get the final result

$$D_{\mathrm{ind}} = \frac{2\pi D\rho\lambda}{R^3}\left\{R(r_m^2 - R^2) + R^3\log(r_m/R) - (r_m^2 - R^2)^{\frac{3}{2}} sin^{-1}\left(\frac{R}{r_m}\right)\right\}.$$

Or, using the definition of λ:

$$D_{\mathrm{ind}} = \frac{2D}{R^3[1 + 2\log\left(r_{max}/r_{min}\right)]}\left\{R(r_m^2 - R^2) + R^3\log(r_m/R) - (r_m^2 - R^2)^{\frac{3}{2}} sin^{-1}\left(\frac{R}{r_m}\right)\right\}.$$

Figure 7c of the main article shows comparison with the simulation results. One can note that the contribution is proportional to the dose D, but not to the LET of the tracks.
